# Ageing of *Plasmodium falciparum* malaria sporozoites alters their motility, infectivity and reduces immune activation in vitro

**DOI:** 10.1186/s12936-024-04946-7

**Published:** 2024-04-19

**Authors:** Roos van Schuijlenburg, Samaneh Azargoshasb, Clarize M. de Korne, Jeroen C. Sijtsma, Sascha Bezemer, Alwin J. van der Ham, Els Baalbergen, Fiona Geurten, Laura M. de Bes-Roeleveld, Severine C. Chevalley-Maurel, Matthias N. van Oosterom, Fijs W. B. van Leeuwen, Blandine Franke-Fayard, Meta Roestenberg

**Affiliations:** 1 Leiden University Center for Infectious Diseases (LUCID), Leiden University Medical Center, Leiden, Netherlands; 2 Interventional Molecular Imaging Laboratory, Department of Radiology, Leiden University Medical Center, Leiden, Netherlands

**Keywords:** Malaria, *Plasmodium falciparum*, Sporozoites, Motility, Immunogenicity

## Abstract

**Background:**

Sporozoites (SPZ), the infective form of *Plasmodium falciparum* malaria, can be inoculated into the human host skin by Anopheline mosquitoes. These SPZ migrate at approximately 1 µm/s to find a blood vessel and travel to the liver where they infect hepatocytes and multiply. In the skin they are still low in number (50–100 SPZ) and vulnerable to immune attack by antibodies and skin macrophages. This is why whole SPZ and SPZ proteins are used as the basis for most malaria vaccines currently deployed and undergoing late clinical testing. Mosquitoes typically inoculate SPZ into a human host between 14 and 25 days after their previous infective blood meal. However, it is unknown whether residing time within the mosquito affects SPZ condition, infectivity or immunogenicity. This study aimed to unravel how the age of *P*. *falciparum* SPZ in salivary glands (14, 17, or 20 days post blood meal) affects their infectivity and the ensuing immune responses.

**Methods:**

SPZ numbers, viability by live/dead staining, motility using dedicated sporozoite motility orienting and organizing tool software (SMOOT), and infectivity of HC-04.j7 liver cells at 14, 17 and 20 days after mosquito feeding have been investigated. In vitro co-culture assays with SPZ stimulated monocyte-derived macrophages (MoMɸ) and CD8^+^ T-cells, analysed by flow cytometry, were used to investigate immune responses.

**Results:**

SPZ age did not result in different SPZ numbers or viability. However, a markedly different motility pattern, whereby motility decreased from 89% at day 14 to 80% at day 17 and 71% at day 20 was observed (p ≤ 0.0001). Similarly, infectivity of day 20 SPZ dropped to ~ 50% compared with day 14 SPZ (p = 0.004). MoMɸ were better able to take up day 14 SPZ than day 20 SPZ (from 7.6% to 4.1%, p = 0.03) and displayed an increased expression of pro-inflammatory CD80, IL-6 (p = 0.005), regulatory markers PDL1 (p = 0.02), IL-10 (p = 0.009) and cytokines upon phagocytosis of younger SPZ. Interestingly, co-culture of these cells with CD8^+^ T-cells revealed a decreased expression of activation marker CD137 and cytokine IFNγ compared to their day 20 counterparts. These findings suggest that older (day 17–20) *P*. *falciparum* SPZ are less infectious and have decreased immune regulatory potential.

**Conclusion:**

Overall, this data is a first step in enhancing the understanding of how mosquito residing time affects* P*. *falciparum* SPZ and could impact the understanding of the *P*. *falciparum* infectious reservoir and the potency of whole SPZ vaccines.

**Supplementary Information:**

The online version contains supplementary material available at 10.1186/s12936-024-04946-7.

## Background

Novel vaccines and drugs are urgently needed to alleviate the burden of disease by malaria and reverse the recent resurgence of cases [1–3]. *Plasmodium falciparum* sporozoites (SPZ), the infective form of malaria, are considered a key target for malaria vaccines and antibodies [4–6]. The relatively low number of SPZ and the fact that these initially remain extracellular in the human host makes them a vulnerable target. SPZ reside principally in the salivary gland of *Anopheles* mosquitoes [7, 8], where they appear 10–14 days post mosquito infected blood meal following sporulation of oocyst in the mosquito midgut wall [7, 8]. Within the salivary gland, SPZ change their infective and metabolic state [9, 10], causing a reduction in the numbers of SPZ per mosquito of about 90% over 4 weeks, with the steepest decline occurring at 25 days post blood meal [11]. In nature, *Anopheles* mosquitoes are thought to survive 10–20 days [12, 13]. A better understanding of the number and infectivity of SPZ in this timeframe may thus help to define the infectious reservoir of SPZ within mosquitoes and may also aid to improve vaccines [14].

Infectivity of SPZ is defined by their ability to actively migrate to and infect liver cells. A relation between motility and infectivity is shown by SPZ surface proteins circumsporozoite protein (CSP) and thrombospondin-related adhesion protein (TRAP) which are both involved during motility and infectivity [15, 16]. On their journey to the liver, SPZ may encounter human immune cells such as macrophages and dendritic cells which can present SPZ antigens and activate CD8^+^ T-cells [17–19]. These primed CD8^+^ T-cells migrate to the liver and are required and capable of recognizing and eliminating infected hepatocytes [19–21]. A dose-dependent increase of CD8^+^ T-cells has been shown in volunteers immunised with SPZ which are attenuated to avoid pathology [22]. These attenuated whole SPZ vaccines are currently undergoing late-stage clinical testing and rely on their preserved metabolic activity which enables them to infect liver cells. To further improve the immunogenicity and efficacy of these vaccines, a better understanding of the infectivity of SPZ at different timepoints after blood meal and ensuing host immune responses is needed.

**Fig. 1:**
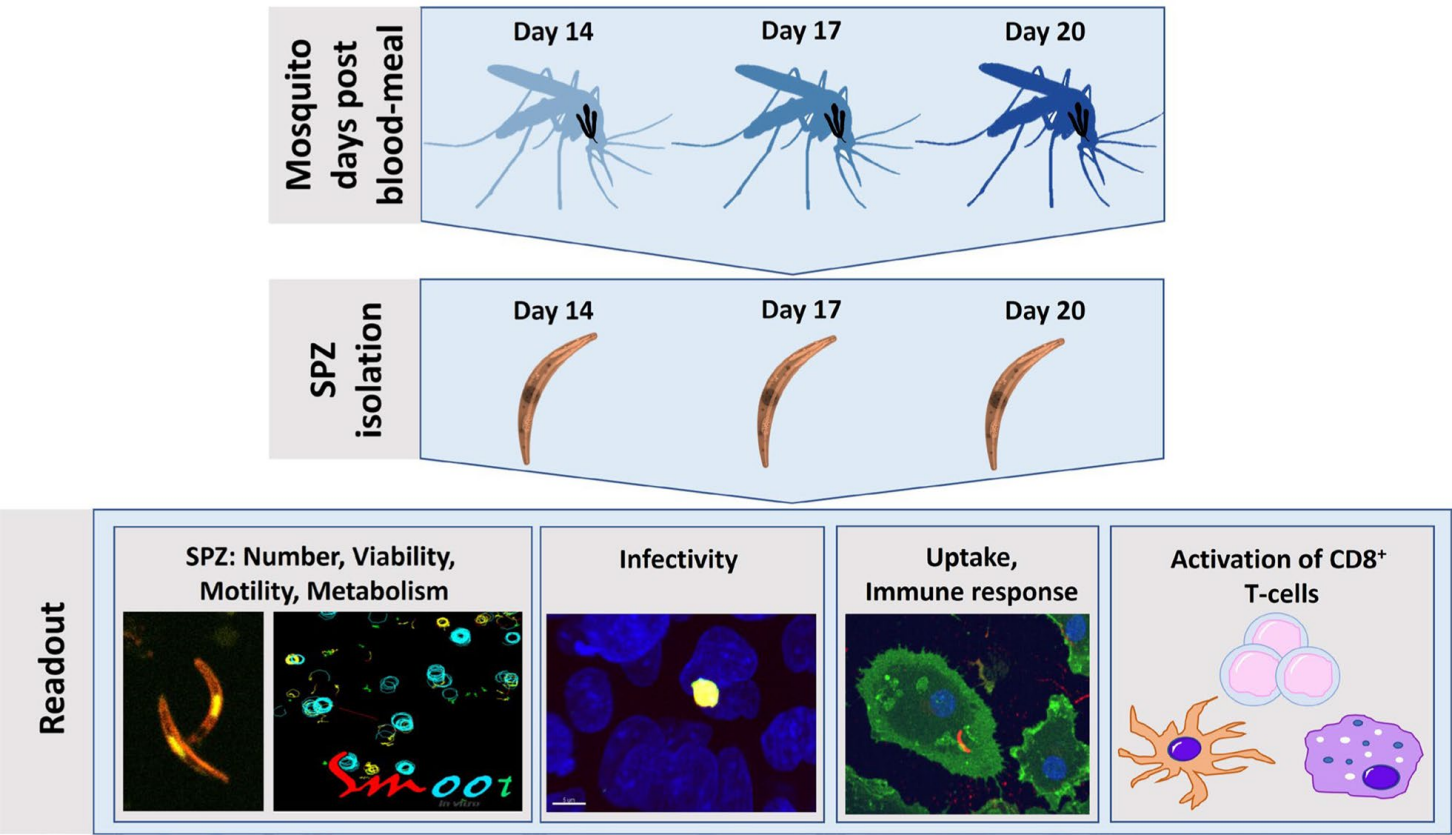
Experimental setup. *Plasmodium falciparum* infected mosquitoes were dissected at day 14, 17 or 20 post blood meal. To determine the number of SPZ, they were counted by light microscopy. Viability was determined by counting after Propidium Iodide staining using a confocal microscope. Motility of SPZ was analysed by traditional gliding assay and automated sporozoite motility orienting and organizing tool (SMOOT) analyses of confocal videos. Metabolism of SPZ was measured by real-time cell metabolic analyses. Infectivity was measured by in vitro infection of HC-04.j7 cells and analysed by immunofluorescence stains and real-time PCR of *P*. *falciparum* targets. Immune responses were assessed by SPZ stimulation of monocyte-derived macrophages (MoMϕs) for assessment of uptake (1 h) by confocal microscopy, phenotype (24 h) by flowcytometry and function (24 h) by ELISA. To investigate the activation of CD8^+^ T-cells, SPZ-stimulated MoMϕs and monocyte-derived dendritic cells (MoDCs) were co-cultured with circumsporozoite protein (CSP) specific CD8^+^ T-cells and analysed by flowcytometry

In summary, previous studies have shown that the age of the SPZ inside the mosquito salivary gland may change their number and infectivity [8–13]. However, little is known about the changes in biology and immunogenicity of aging SPZ at early timepoints post blood meal which seem to be most relevant to the infectious reservoir in endemic areas. This study thus sets out to investigate the changes in SPZ as they age from day 14 to day 20 post blood meal. Alteration in motility, viability, metabolism, infectivity and immune response in vitro was analysed (Fig. 1).

## Methods

### Parasite culture

*Anopheles stephensi* mosquitoes were infected with *P. falciparum* wild-type NF54 [23–25] infected blood through membrane feeding as previously described [26]. Salivary glands of infected and uninfected mosquitoes were dissected 14, 17 or 20 days post blood meal in RPMI + 10% fetal calf serum (FCS), and homogenized to extract SPZ from the glands [27].

### Sporozoite numbers and viability assessment

SPZ were counted in a Bürker chamber using phase-contrast. After counting, an estimated 15,000 SPZ/well were stained for 15 min (min) at room temperature with propidium iodide at a 1:25 dilution in RPMI + 10% FCS (Thermo Fisher Scientific). The plates were centrifuged for 3 min at 300 × g and assessed by confocal microscopy using Dragonfly 500 spinning disk (Leica Microsystems, Wetzlar). Live and dead SPZ were counted using the Imaris software (version 9.8, Oxford).

### Sporozoite gliding assay

Gliding assays were performed as previously described [28–30]. Briefly, 96 wells optical glass bottom plates were coated with 10 µg/mL monoclonal anti circumsporozoite protein (anti-CSP) 3SP2 (courtesy of Dr. M. McCall Radboud UMC) in phosphate buffered saline (PBS) and incubated overnight at room temperature (RT). After washing, 15,000 SPZ in 100 µl RPMI + 10% FCS were added to each well. Plates were centrifuged for 3 min at 1200 rpm RT and incubated for one hour at 37 °C + 5% CO_2_ after which SPZ were fixed by 3.7% paraformaldehyde (PFA; Sigma Aldrich, St Louis, MO, USA) for 15 min at RT. Subsequently, plates were blocked with 10% FCS/PBS for 15 min at RT, stained with 10 µg/mL primary monoclonal anti-CSP 3SP2 in PBS for 45 min at RT, and stained with secondary antibody goat anti-mouse AF488 (Invitrogen) 1:500 in PBS and incubated for 45 min in the dark at RT. Between each step the wells were gently washed 3 times with PBS. Each well was measured by confocal microscopy (Leica Microsystems, Wetzlar). Each condition was performed in duplicate. A total number of 10 pictures of each well was analysed and the tracks were counted by two independent readers.

### Motility assessment by automated video analyses

Motility of SPZ were analysed using the custom-made SMOOT software, as previously described [30, 31]. Briefly, SPZ were diluted to 15.000 spz/100 µl RPMI + 10% FCS and 100 µL/well was added to 96 wells optical glass bottom plate. 200 nm Cy5-methyl-methyl (Cy5M_2_) was used for staining of the SPZ as described previously [30, 31]. The plate was centrifuged for 3 min at 1200 rpm RT. Directly after centrifugation 200 frames were measured by Leica TCS SP5 (Leica Microsystems, Wetzlar) at 28 °C. Each condition was measured in triplicate. Kinematic parameters were extracted using SMOOT software.

### SPZ real-time cell metabolic analyses

The wells of a XFe96 well Seahorse plate (Agilent) were coated with 10 µg/mL 2A10 aCSP (BEI resources) in PBS and incubated over night at room temperature. The following day, SPZ were obtained from salivary glands in XF medium RPMI (Sigma Aldrich, St Louis, MO, USA, R6504) supplemented with 10 mM glucose (Sigma Aldrich, St Louis, MO, USA, G8644) 5% FCS and 1% l-glutamine (20 µM). The anti-CSP coated wells were washed with PBS. To each well 180,000 SPZ of 14 days or 20 days post blood meal were added. Before measuring, the plate was spun down for 3 min at 1200 rpm and rested for 15–30 min at 37 °C non-CO_2_. Injected compounds were diluted in XF media to a concentration of 2 μM for oligomycin (Cayman, 11342), 1.5 μM for FCCP (Sigma, C2920), 0.75 μM for rotenone (Sigma, 557368) and 0.75 μM for antimycin (Sigma, A8674). Each condition was measured in quadruple. As negative control salivary glands extract (SGE) of comparable number of uninfected mosquitoes were measured and subtracted using the manufacturer’s Wave Seahorse software.

### Hepatocyte infection assay

A 96 wells optical glass bottom plate was coated with 50 µg/mL collagen type I (Corning, USA) in PBS for 30 min at RT. The wells were gently washed with PBS and 40.000 HC-04.j7 cells per well (kindly provided by Dr. Rhoel Dinglasan) were plated in 200 µl DMEM + 5% FCS supplemented with 1% penicillin/streptomycin and incubated overnight at 37 °C + 5% CO_2_. The following day, the medium was refreshed with 100 µl DMEM + 5% FCS supplemented with 1% penicillin/streptomycin, 15 mM glucose (Thermo Fisher Scientific, Waltham, MA, USA) and 0.01 mg/mL insulin, 5.5 µg/mL transferrin and 0.67 µg/mL selenium (Thermo Fisher Scientific, Waltham, MA, USA). SPZ were extracted from salivary glands as previously described and diluted to 60,000 SPZ/100 µl DMEM + 5% FCS supplemented with 1% penicillin/streptomycin, 15 mM glucose (Thermo Fisher Scientific, Waltham, MA, USA) and 0.01 mg/mL insulin, 5.5 µg/mL transferrin and 0.67 µg/mL selenium (Thermo Fisher Scientific, Waltham, MA, USA). Heat-killed SPZ, killed by incubation at 100 °C for 30 min, were used as a negative control. Medium was removed from the HC-04.j7 cells and 100 µl SPZ/heat-killed SPZ/SGE from 14, 17 or 20 days post blood meal were added. The plate was centrifuged for 3 min at 1200 rpm and incubated at 37 °C + 5% CO_2_. At day 2 post infection (pi) the medium was refreshed with 100 µl 37 °C warm DMEM + 5% FCS supplemented with 1% penicillin/streptomycin, 15 mM glucose (Thermo Fisher Scientific, Waltham, MA, USA) and 0.01 mg/mL insulin, 5.5 µg/mL transferrin and 0.67 µg/mL selenium (Thermo Fisher Scientific, Waltham, MA, USA). At day 3 pi, the cells were fixed with 3.7% paraformaldehyde (PFA; Sigma Aldrich, St Louis, MO, USA) in PBS for 15 min at RT. After the permeabilization with 0.5% Triton (Thermo Fisher Scientific, Waltham, MA, USA) in PBS for 20 min the cells were washed and blocked with 10% FCS in PBS for 30 min, and stained with primary antibodies HSP70 1:500 (Thermo Fisher Scientific, Waltham, MA, USA) and GAPDH 1:1000 (The European malaria reagent repository, Edinburgh) in PBS overnight at 4 °C. The following day the cells were washed 3 times with PBS and stained with secondary antibodies anti-rabbit AF594 (Invitrogen) 1:500 and anti-mouse AF488 (Invitrogen) 1:500 in PBS for 1 h in the dark. The nucleus of the cells was stained with Hoechst 33342 (Sigma) 1:200 for 30 min in the dark. The cells were washed 3 times and 100 µl PBS was added to each well. The wells were analysed by ImageXpress confocal (Molecular Devices, LLC, San Jose USA). The HSP70 and GAPDH positive cells were counted by high-content image acquisition and analysis software MetaXpress (Molecular Devices, LLC, San Jose USA).

In addition, infectivity of HC-04.j7 was assessed by qPCR. At 72 h pi the cells were washed twice with PBS, and 100 µl RLT buffer (Qiagen) was added. The cells were lysed within 20 min and stored at − 80 °C. To assess SPZ infection by PCR, the 18S and EXP1 ribosomal RNA (rRNA) levels were determined. RNA was isolated from the cell cultures using the RNeasy kit (Qiagen) and cDNA was generated using random hexamer primers (Promega) and Superscript™ III (Thermo Fisher) according to manufacturer’s instructions. Amplification reactions of each cDNA sample were performed in PCR plates (hard-shell PCR plate; Bio-Rad), in a volume of 10 μl containing 5 μl PCR buffer (GoTaq qPCR master mix; Promega), 1 μl RNase-free milliQ water, 2 μl primer mix (for 18S: forward primer: Plasmo Plu3F 5ʹ-GCTCTTTCTTGATTTCTTGGATG-3ʹ (0.2 µM; Integrated DNA Technologies, Inc.); reverse primer: Plasmo Plu3R 5ʹ- AGCAGGTTAAGATCTCGTTCG-3ʹ [0.2 µM; Integrated DNA Technologies, Inc. and for EXP1 forward primer: 5ʹ-GCTAACCCAGATGCTGATTCTG-3ʹ (0.2 µM; Integrated DNA Technologies, Inc.) and reverse primer: 5ʹ-GTGTTCAGTGCCACTTACGAGG-3ʹ (0.2 µM; Integrated DNA Technologies, Inc.)] and 2 μl of the cDNA sample. Amplification was performed 10 min at 95 °C followed by 50 cycles of 10 s at 94 °C, 15 s at 60 °C, and 30 s at 72 °C. Amplification, detection, and analysis were performed with the CFX96TM real time PCR detection system (Bio-Rad CFX96 real time qPCR thermocycler).

### Monocyte-derives macrophages (MoMɸs) and monocyte-derives dendritic cells (MoDCs)

Monocytes were isolated from whole blood of healthy volunteers using CD14^+^ MACS isolation (miltenyi Biotec, Bergisch Gladback, Germany), and differentiated into MoMɸs using 20 ng/mL macrophage colony stimulating factor (M-CSF; Biolegend, San Diego, CA, USA) [32] or MoDCS using 20 ng/mL granulocyte–macrophage colony-stimulation factor (GM-CSF; Biosource/invitrogen, Carlsbad, CA, USA) and 0.86 ng/mL human rIL4 (R&D systems, Minneapolis, MN, USA) in RPMI + 10% FCS supplemented with penicillin/streptomycin [33]. On day 6, MoMɸs or MoDCs were harvested, counted and 50,000 cells/well for uptake, and 100,000 cells/well for flowcytometry were plated in flat bottom 96 wells plate and rested for overnight at 37 °C + 5% CO_2_.

### Sporozoite uptake analysis

MoMɸs were stimulated with 1:1 ratio SPZ for one hour at 37 °C + 5% CO_2_. Next, the cells were fixed by 3.7% paraformaldehyde (PFA; Sigma Aldrich, St Louis, MO, USA) 15 min at RT. Each well was washed 3 times with PBS. After permeabilization with permeabilization buffer (Affymetritimes, Santa Clara, CA, USA) the cells were first stained with 10 µg/mL primary monoclonal anti-CSP 3SP2 in PBS for 45 min, washed and then incubated with secondary antibody goat anti mouse AF647 (Invitrogen) 1:1000 in PBS for 45 min in the dark. Cell nuclei were stained with Hoechst 33342 1:200 for 20 min in the dark. Finally, the cell membrane was stained with CellBrite (Biotium, Hayward, CA, USA) 1:200 in PBS for 10 min in the dark. Between each step the cells were gently washed 3 times with PBS. Uptake of SPZ by MoMɸs was measured by Dragon fly confocal microscopy (Leica Microsystems, Wetzlar). Number of cells was counted by ImageJ and number of SPZ taken up by MoMɸs was counted by using the Imaris software (version 9.8, Oxford) by two independent readers. MoMɸs with a total of 5 donors (provided by Sanquin Amsterdam, NL) were stimulated over two separate experiments with different mosquito batches and analysed in duplicate.

### MoMɸs flowcytometry and cytokine measurements

In addition, the phenotype of MoMɸs was assessed by flow cytometry and cytokine measurements. For this purpose, MoMɸs were stimulated with 1:1 ratio SPZ, salivary gland extract (SGE) as a negative control, medium as background or 100 ng/mL lipopolysaccharide (LPS) as a positive control for 24 h at 37 °C + 5% CO_2_. After 24 h supernatant was collected and the cells were harvested, washed with PBS and stained for CD209 (DCN46; BD Bioscience), CD163 (9332, BD Bioscience), PD-L1 (MIH1; BD Bioscience) ILT3 (zm4.1; Biolegend), CD206 (15-2; Biolegend), CD80 (L307.3; BD Bioscience), CD25 (2A3; BD Bioscience), aqua live/dead staining (Thermofisher), Fc-block (BD Bioscience) in PBS for 30 min at 4 °C and measured by flow cytometry using LSR Fortessa (BD bioscience, San Jose, CA, USA). The data was analysed by FlowJo version 10.8 (FlowJo LLC, Ashland, OR, USA). After 24 h stimulation of MoMɸs with SPZ from day 14, 17 and 20 post blood meal, and controls (LPS, SGE, medium) the supernatants were diluted 1:5 (IL6) or 1:2 (IL10 and TNF) and analysed by standard ELISA (Sanquin, The Netherlands).

### CSP specific CD8^+^ T-cell co-culture

CSP specific CD8^+^ T-cells (kindly provided by Prof. G. Corradin, University of Lausanne, Switzerland) were cultured as described previously [19]. Briefly, expanded CSP specific CD8^+^ T-cells were thawed and cultured with irradiated Human Leukocyte Antigens a2 (HLA-A*02) positive Peripheral Blood Mononuclear Cells (PBMCs) (Sanquin, Amsterdam), 5 ng/mL human rIL-15 (Miltenyi Biotec), 5 ng/mL human rIL-7 (Miltenyi Biotec) and aCD3/28 Dynabeads (Thermo Fisher Scientific) in IMDM + 10% heath inactivated human serum. After two days, 50 U/mL human rIL-2 (Miltenyi Biotec) was added. When needed the cells were split and 100 U/mL IL-2 was added. The cells were used 10 days after expansion.

MoDCs and MoMɸs of HLA-A*02 donors were stimulated for 24 h at 37 °C + 5% CO_2_ with 1:1 ratio live SPZ, SGE or dead SPZ, killed by 3 times snap freezing in liquid nitrogen. After 24 h the cells were counted and 10,000 SPZ stimulated MoMɸs and 10,000 SPZ stimulated MoDCS were co-cultured with 40,000 CSP specific CD8^+^ T-cells in a U bottom 96 wells plate and incubated o/n at 37 °C + 5% CO_2_. After 4 h 3 µg/mL brefeldin A (Thermofisher) was added to the culture. The following day, the cells were harvested and washed with PBS and stained with aqua live/dead (Thermofisher) for 20 min at RT. The cells were washed and fixed with 3.7% paraformaldehyde (PFA; Sigma Aldrich, St Louis, MO, USA) in PBS for 15 min at RT. The cells were washed twice and stained with CD137 PE (Biolegend), CD3 APC-ef780 (eBioscience), IFNy HV450 (Biolegend), Perforin PERCP-ef710 (eBioscience), Granzyme A PECy7 (Biolegend), Granzyme B APC (Biolegend) for 30 min at 4 °C and measured by flow cytometry using LSR Fortessa (BD bioscience, San Jose, CA, USA) and analysed by FlowJo version 10.8 (FlowJo LLC, Ashland, OR, USA).

### Statistical analysis

Data was analysed using SPSS. Comparison between two independent data groups were made by Mann Whitney U test (for nonparametric data) or Fishers exact. p < 0.05 was considered statistically significant. PLS-DA performed with R studio, tSNE with Matlab.

## Results

### Number of SPZ and viability

**Fig. 2:**
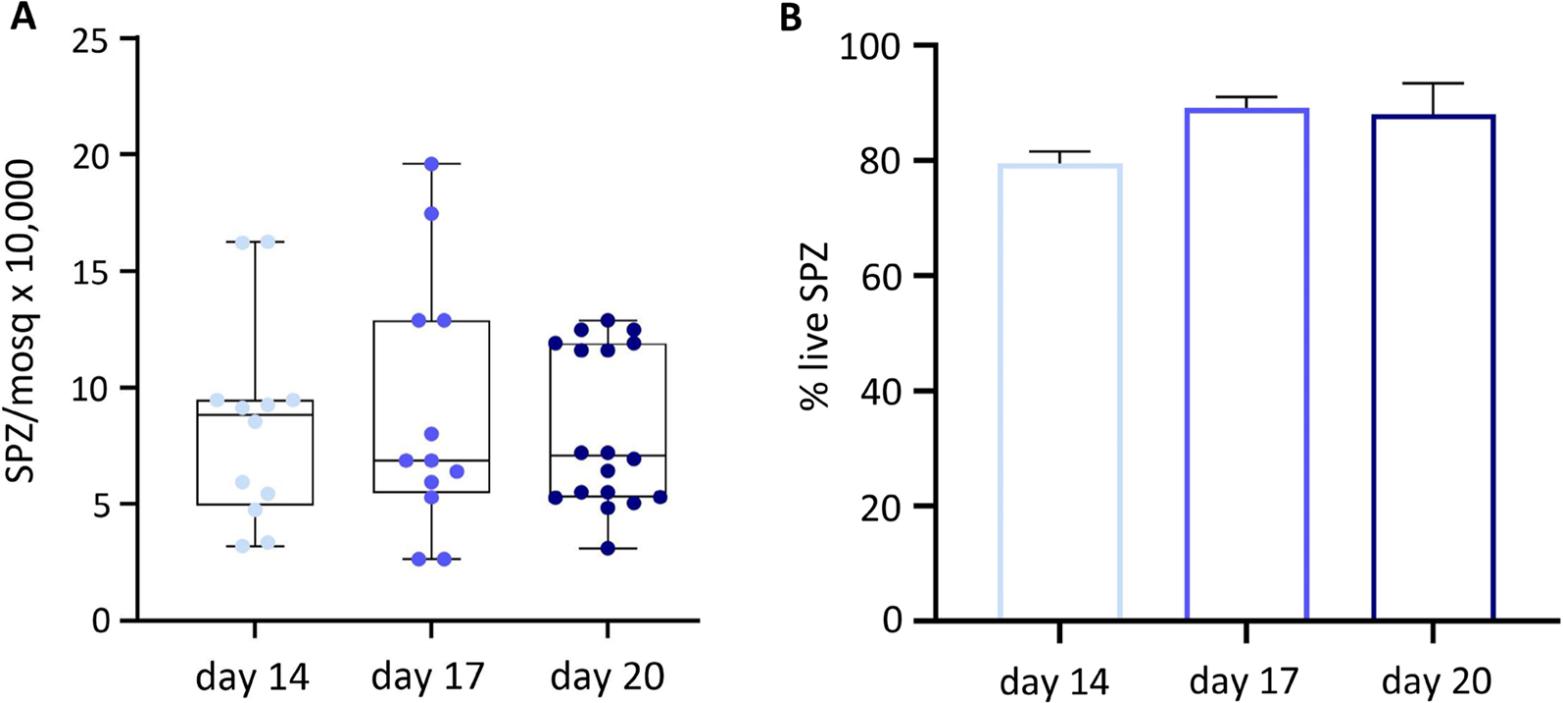
Number and viability of SPZ. **A** Average number of SPZ per mosquito after 14 days (light blue), 17 days (blue) or 20 days (dark blue), post blood meal (A). **B** Percentage of live and dead SPZ 14 days (light blue), 17 days (blue) or 20 days (dark blue), post blood meal. Analysis using Mann Whitney U test, Fishers exact. *p ≤ 0.05, **p ≤ 0.005, ***p ≤ 0.0005 and ****p ≤ 0.0001

First the number of salivary gland SPZ was determined by microscopic counting. There were no significant differences in the number of SPZ per mosquito dissected at day 14 (mean 8.4; SD 4.33) day 17 (mean 8.9; SD 5.5) or day 20 (mean 8.2; SD 3.4) post blood meal, between batches (Fig. 2A), nor within the same batch of mosquitoes (Additional file 1). Subsequently, the percentages of live and dead SPZ using the viability marker propidium iodide were determined. At day 14 post blood meal, there were no significant differences in proportion of live SPZ (mean 79.5%; SD 2.1) as compared to day 17 (mean 89.1%; SD 1.9) or day 20 SPZ (mean 88.1%; SD 5.4. Figure 2B).

### Sporozoite motility

**Fig. 3:**
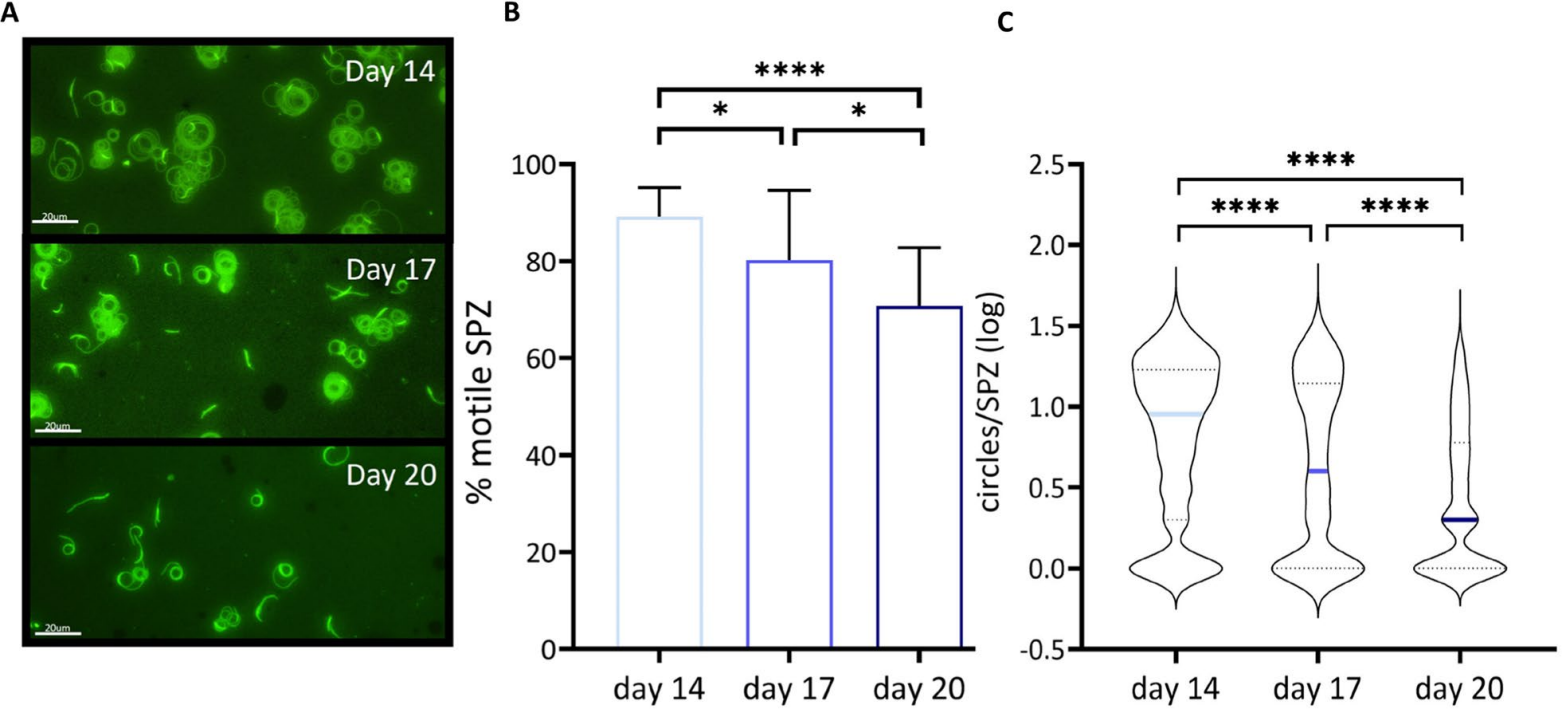
SPZ motility by immunofluorescent staining of tracks. **A** Representative image of SPZ tracks obtained from SPZ harvested at day 14, day 17 and day 20 post blood meal.** B** Percentage (%) of moving SPZ and stationary SPZ at day 14 (light blue), day 17 (blue) and day 20 (dark blue). **C** Distance travelled by motile SPZ determined by counting circles, n = 3 with day 14 (light blue) 794 SPZ tracks, day 17 (blue) 469 SPZ tracks and day 20 (dark blue) 775 SPZ tracks. Analysis using Mann Whitney U test. *p ≤ 0.05, **p ≤ 0.005, ***p ≤ 0.0005 and ****p ≤ 0.0001

Next the motility of the SPZ at 14, 17 or 20 days post blood meal by immunofluorescence staining of SPZ tracks (gliding assay) was analysed. There was a significant decrease in percentage of motile SPZ harvested at later timepoints (day 14: mean 89.1%; SD 6.0, day 17: mean 80.2%; SD 14.4, day 20: mean 70.8%; SD 12.0, day 14 compared with day 20: p ≤ 0.0001, Fig. 3B). In addition, motile SPZ on average showed a decrease in the distance travelled over time at later timepoints, as measured by the number of circles counted per sporozoite (day 14: mean log 0.9; SD 1.0, day 17: mean log 0.6; SD 1.2, day 20: mean log 0.3; SD 0.8, p ≤ 0.0001, Fig. 3C).

In addition to a traditional assessment by gliding assay, SPZ motility using purpose-built tracking software (SMOOT) which allows us to extract kinetic features in more detail was analysed. The following parameters were extracted: number of frames per track, velocity, angular dispersion and straightness index. When analysing motility features for each of the SPZ ages separately, a significant decrease in number of frames between SPZ from 14 days post blood meal (mean 50.8, Additional file 2A) as compared with 17 days (mean 28.6) and 20 days post blood meal (mean 31.9) were observed, confirming that day 14 SPZ on average have longer tracks. In addition, an increase in velocity (day 14: mean 1.2, day 17: mean 1.6, day 20 mean: 1.6, Additional file 2B) angular dispersion (day 14: mean 0.5, day 17: mean 0.6, day 20 mean: 0.7, Additional file 2C) and straightness index (day 14: mean 0.3, day 17: mean 0.4, day 20 mean: 0.4, Additional file 2D) for 14 days post blood meal SPZ was found, indicating slower and more chaotic movement patterns for the day 14 SPZ.

**Fig. 4:**
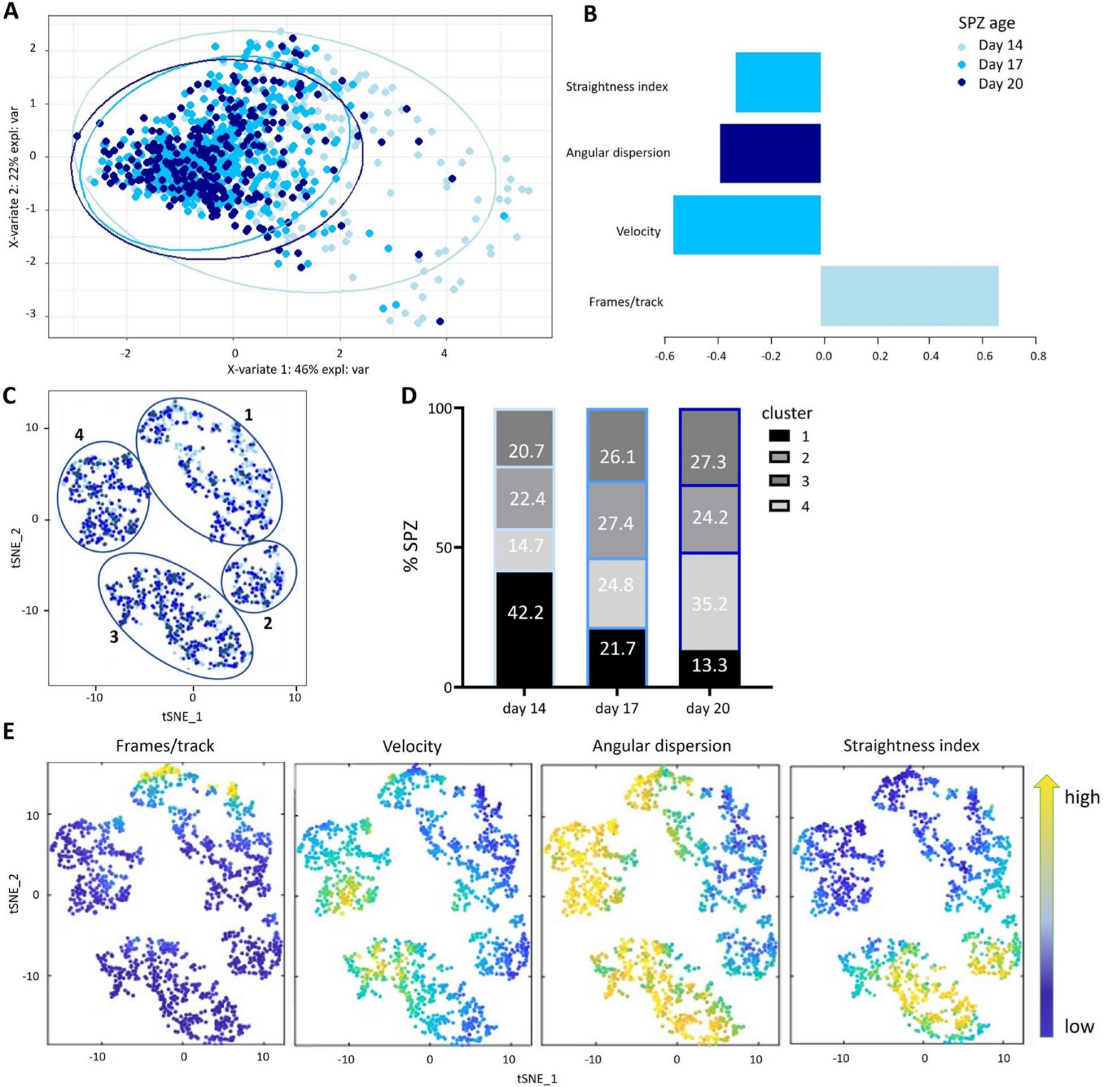
Motility by SMOOT. **A** PLS-DA of day 14 SPZ (light blue), day 17 SPZ (blue) and day 20 SPZ (dark blue). **B** Loading plot for PLS-DA. **C** tSNE of day 14 SPZ (light blue), day 17 SPZ (blue) and day 20 SPZ (dark blue). **D** Percentage of SPZ from each timepoint per cluster plotted in stacked bars. **E** Features measured and analysed by SMOOT: frames, velocity, angular dispersion and straightness index. PLS-DA performed with R studio, tSNE with Matlab

Day 14 SPZ could be separated from older SPZ by partial least squares-discriminant analysis (PLS-DA) analysis (Fig. 4A). The PLS-DA loading plot (Fig. 4B) showed that the length of the SPZ track (frames/track) positively discriminates day 14 SPZ from 17 and 20 SPZ. Additionally, straightness index and velocity of day 17 SPZ and angular dispersion of day 20 SPZ are also kinetic features distinguishing day 14 SPZ from their counterparts. Using T-Distributed Stochastic Neighbour Embedding (tSNE) a clear clustering into 4 motility clusters (Fig. 4C–E) was observed. SPZ of all ages were almost represented equally in cluster 2 and 3 (Fig. 4D). Cluster 1 was overrepresented in young day 14 SPZ with 42.2% of events, as compared to 22.4% in cluster 2, 20.7% in cluster 3 and 14.7% in cluster 4 (Fig. 4D). Cluster 1 SPZ showed an increased number of frames, a lower velocity, and a slightly lower angular dispersion and straightness index as compared to the other clusters, suggesting that SPZ in this cluster move longer, slower and more chaotic. Within cluster 1 almost half (49%) were young day 14 SPZ and only 17% old day 20 SPZ.

To investigate whether the motility patterns are unique to viable SPZ, the differences in oxygen consumption rate (OCR) and extracellular acidification rate (ECAR) metabolic states (Additional file 3A–D) were investigated. There were no significant differences when SPZ age (OCR, AUC Day 14: mean 68.4 SD 128; Day 20: mean 23.16 SD 32, ECAR, AUC Day 14: mean 58.34; SD 60, Day 20: mean 71.97; SD 58, Additional file 3A–C). An increase in ECAR is known as a metabolic pathway shown active in cells undergoing stress [34], and shows that day 20 SPZ are not consistently more stressed than day 14 SPZ, although these data were highly variable. When the basal energy metabolism was investigated, which can be calculated by dividing OCR by ECAR AUC score, there were no differences observed in basal energy when SPZ age (Day 14: mean 1.77, Day 20: mean 0.32, Additional file 3D). These data suggest that the metabolic activity of day 20 SPZ is comparable with day 14 SPZ and thus OCR and ECAR metabolic changes do not underly the different motility patterns between day 14 and 20 SPZ.

Collectively, these data show that SPZ from 14 days post blood meal move more, longer, with a lower velocity, more chaotic and more in circles compared with SPZ at 17 and 20 days post blood meal even though they share comparable metabolic activity.

### Infectivity

**Fig. 5:**
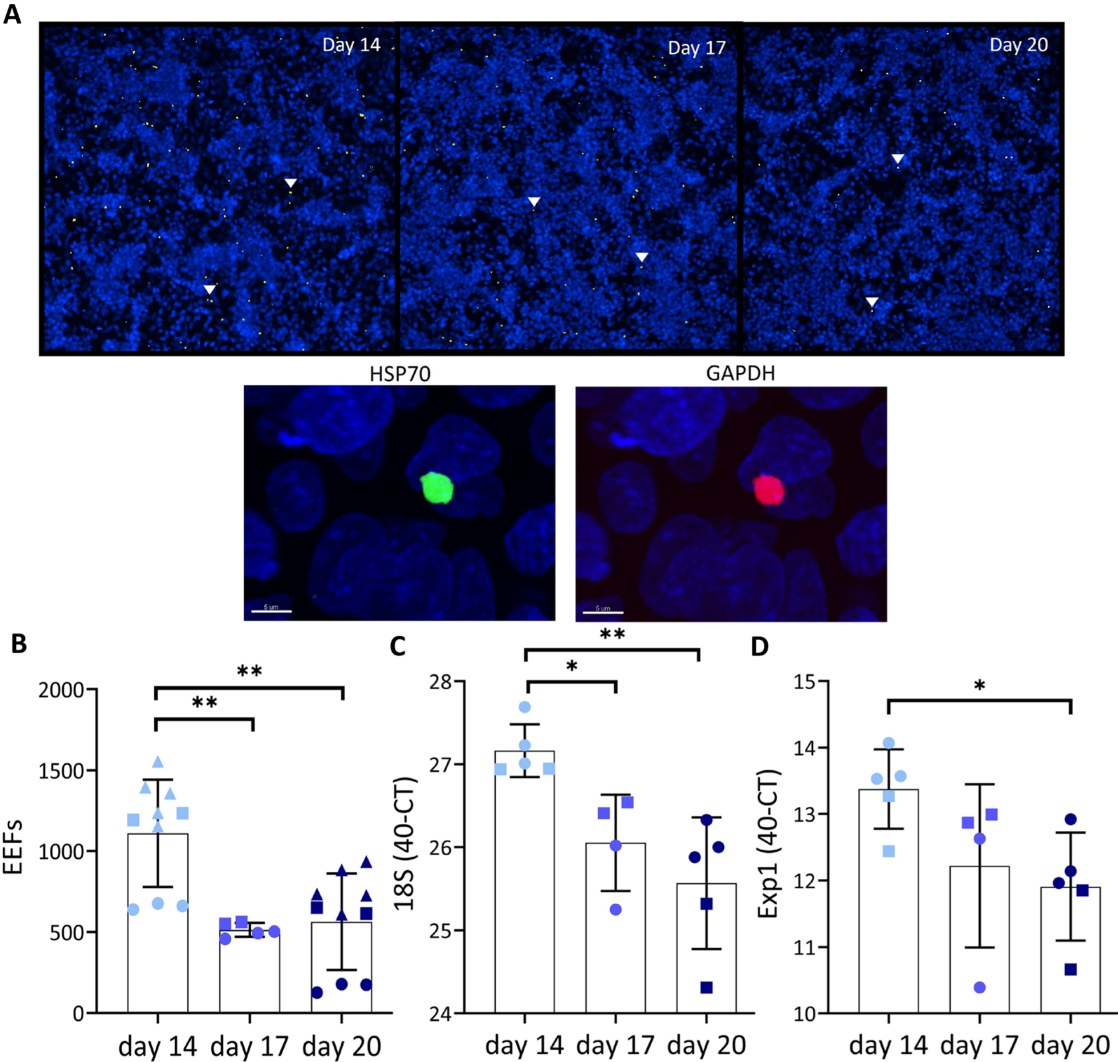
Infectivity of SPZ to HC-04.j7 hepatocytes. **A** Representative image of overlay 3 × 3 fields by ImageXpress of liver schizonts in HC-04.j7 cells, 72 h post infection with day 14, 17 and 20 SPZ. HC-04.j7 nucleus in blue, liver schizont double positive, HSP70 in green, GAPDH in red and overlay in yellow. **B** Number of HSP70 and GAPDH expressing exoerythrocytic forms (EEFs) 72 h after infection with day 14 SPZ (light blue), day 17 SPZ (blue) and day 20 SPZ (dark blue).** C** 18S PCR of infection assay of HCO4.j7 cells infected with SPZ at day 14 (light blue), day 17 (blue) and day 20 (dark blue). **D** Exp1 PCR of infection assay with HCO4.j7 cells. Analysis using Mann Whitney U test. Individual experiments indicated with different shapes (circle, square or triangle). *p ≤ 0.05, **p ≤ 0.005, ***p ≤ 0.0005 and ****p ≤ 0.0001

To analyse the effect of the SPZ age on infectivity, a hepatocyte infection assay in HC-04.j7 cells was performed (Fig. 5A). In the number HC-04.j7 cells infected with SPZ at a later age a 2.2 fold decrease was found, e.g. day 17 SPZ (mean 513; SD 43. p = 0.0007. Figure 5B). Additionally, day 20 SPZ (mean 563; SD 298. p = 0.004. Figure 5B) show a twofold decrease in the number of exoerythrocytic forms (EEFs) as compared with day 14 SPZ (mean 1110; SD 332. Figure 5B). We further confirmed infectivity by 18S PCR (Day 14: mean 40-Ct 27; SD 0.3. Day 17: mean 40-Ct 26; SD 0.6. Day 20: mean 40-Ct 26; SD 0.8. Day 14 compared with day 17 p = 0.02, Day 14 compared with day 20 p = 0.008. Figure 5C) and EXP1 PCR (Day 14: mean 40-Ct 13; SD 0.6. Day 17: mean 40-Ct 12; SD 1.2. Day 20: mean 40-Ct 12; SD 0.8. Day 14 compared with day 20 p = 0.02. Figure 5D). In conclusion, the decreased motility of SPZ harvested at a later age correlates with a decrease in hepatocyte infectivity of SPZ in vitro.

### SPZ immunogenicity

**Fig. 6:**
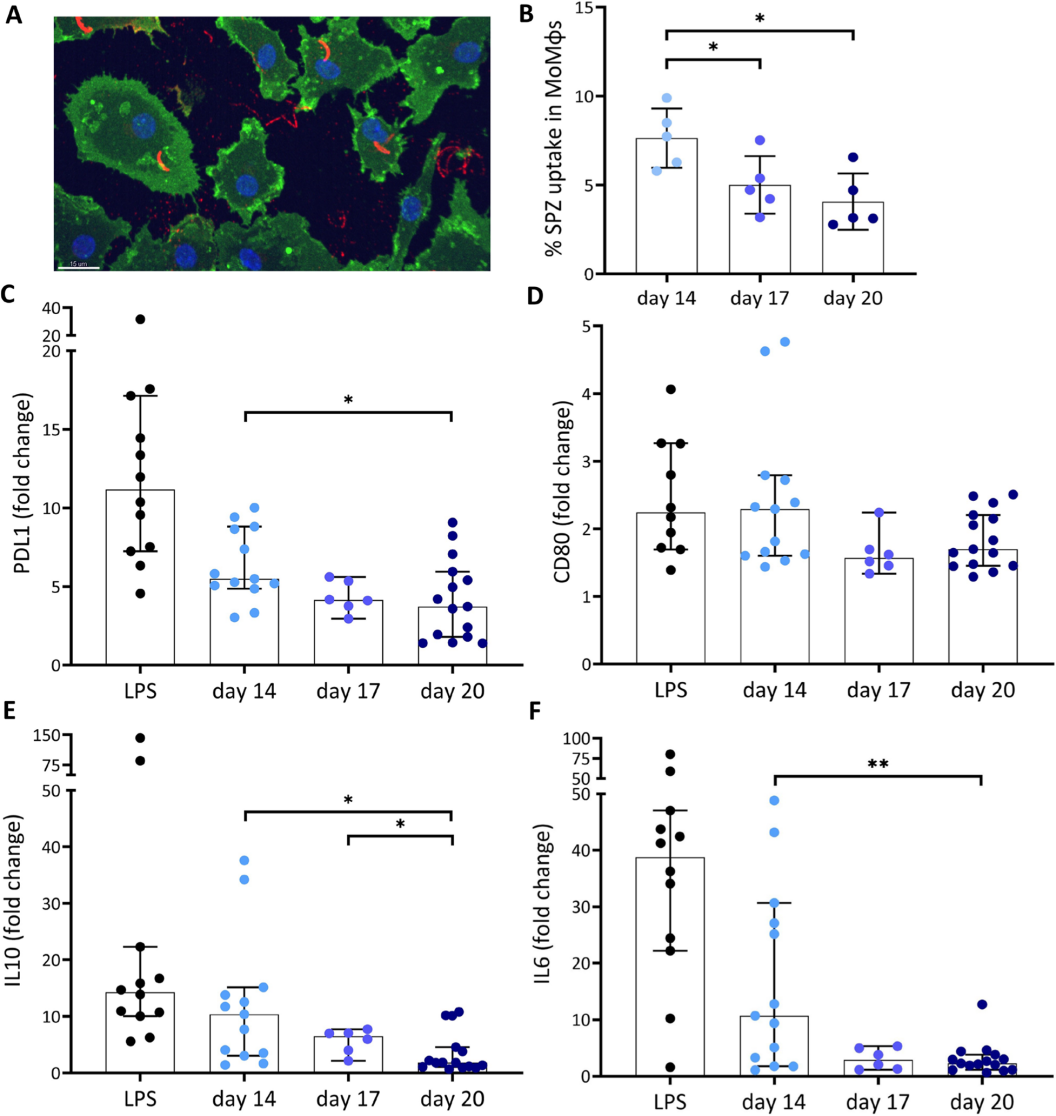
Immune response. **A** Confocal microscopy image of SPZ uptake by MoMɸs. MoMɸs membrane in green, nucleus in blue and SPZ in red. **B** Percentage of *P*. *falciparum* SPZ uptake by MoMɸs of day 14 (light blue), day 17 (blue) and day 20 (dark blue) SPZ. **C** Expression of PD-L1 after stimulation with lipopolysaccharide (LPS) as a positive control (grey), and SPZ used at 14 days (light blue), 17 days (blue) and 20 days (dark blue) post blood meal. Data shown as median fluorescence intensity (MFI) fold change relative to medium stimulated control. **D** Expression of CD80 after stimulation with lipopolysaccharide (LPS) as a positive control (black), and SPZ at 14 days (light blue), 17 days (blue) and 20 days (dark blue) post blood meal. Data shown as median fluorescence intensity (MFI) fold change relative to medium stimulated control. **E** Production of IL-6 by MoMɸs after 24 h in pg/mL, fold change relative to medium stimulated control. **F** production of IL-10 by MoMɸs after 24 h. Day 14 N = 13, Day 17 N = 6, Day 20 N = 15. Analysis using Mann Whitney U test. *p ≤ 0.05, **p ≤ 0.005, ***p ≤ 0.0005 and ****p ≤ 0.0001

Having shown a decrease in SPZ motility and infectivity with SPZ age, their immunogenicity by looking at phagocytic uptake of SPZ by MoMɸs and subsequent phenotypic and functional responses was investigated next. Despite the fact that older SPZ move less, a significant decrease in uptake of these SPZ by MoMɸs was found (Day 14: mean 7.6%; SD 1.7. Day 17: mean 5.0%; SD 1.6. Day 20 mean 4.1%; SD 1.6. Figure 6A, B). In parallel, the surface marker expression of PD-L1 and CD80 MoMɸs after phagocytosis of SPZ decreased when harvested at later timepoints, e.g. PD-L1 expression decreased from MFI (mean fluorescence intensity) fold change 6.3 (SD 2.3, Fig. 6C) of day 14 SPZ to MFI fold change 4.3 (SD 1.0) at day 17 and MFI fold change 4.1 (SD 2.6) at day 20 (p = 0.02). CD80 expression also decreased somewhat from mean 2.4 (SD 1.1. Figure 6D) at day 14 to mean 1.6 (SD 0.3) and mean 1.8 (SD 2.6) at day 17 and day 20 respectively, although these changes were not significant. With a decrease in surface marker expression, MoMɸs also produced significantly less of the pro-inflammatory cytokine IL-6 (p = 0.005) and regulatory cytokine IL-10 (p = 0.009) between SPZ harvested at 14 days post blood meal and 20 days post blood meal (Fig. 6E, F). Interestingly, other phenotype markers and functional cytokines such as pro-inflammatory CD163 (p = 0.35), anti-inflammatory CD209 (p = 0.84) and pro-inflammatory cytokine TNF (p = 0.75) did not show any difference in expression nor production after stimulation with SPZ harvested at day 14 as compared with day 20 (Additional file 4A–C). These data suggest that the differences in immunogenicity are not caused by the differences in SPZ uptake but are marker and cytokine specific.

**Fig. 7:**
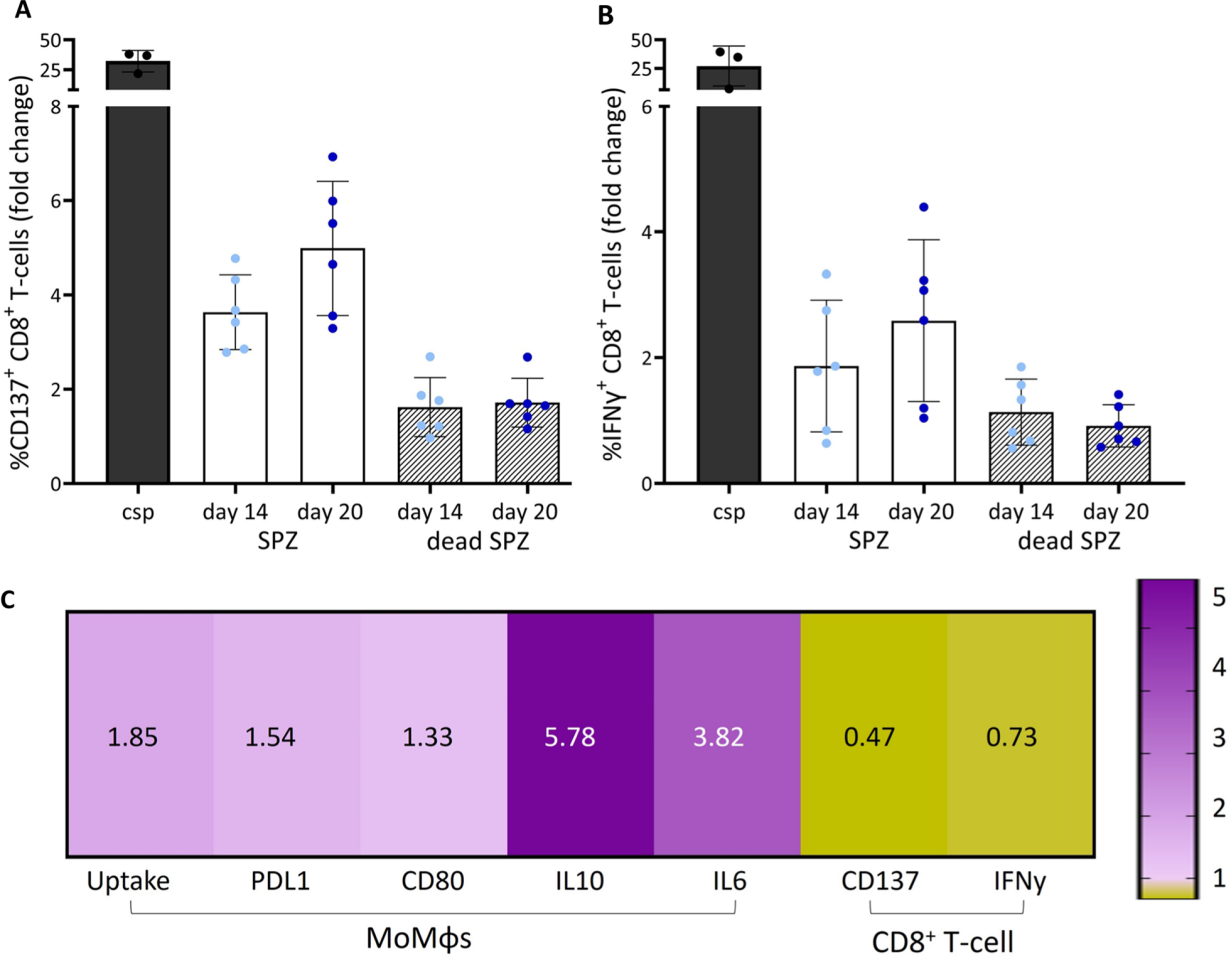
CD8^+^ T-cell response and immunogenicity. **A** Percentage of CD137^+^ CD8^+^ T-cells. CSP (black), day 14 SPZ (light blue), day 20 SPZ (dark blue), day 14 and 20 dead SPZ (grey striped bar). Data shown as fold change relative to medium stimulated control. **B** Percentage of IFNγ^+^ CD8^+^ T-cells. **C** Ratio of day 14 divided by day 20. In purple ratio score above 1, day 14 SPZ show an increased pattern compared with day 20 SPZ. In yellow ratio score below 1, day 14 SPZ show a decreased pattern compared with day 20 SPZ. Calculated for MoMɸs experiments: uptake, expression of PDL1 and CD80 and cytokine production of IL10 and IL6, and for CD8^+^ T-cell co-culture activation markers CD137 and IFNγ. Analysis using Mann Whitney U test. *p ≤ 0.05, **p ≤ 0.005, ***p ≤ 0.0005 and ****p ≤ 0.0001

Because of the mixed expression of both pro- (CD80) and anti-inflammatory (PDL-1) markers and cytokines (IL-6, IL-10), the functional consequences of the changed surface marker expression and cytokine secretion were investigated. MoMɸs and moDCS were stimulated with SPZ of 14 days and 20 days post bloodmeal and co-cultured with CD8^+^ T-cells specific to an epitope sequence of the surface circumsporozoite protein (CSP) of SPZ as previously described [19]. Interestingly, a trend towards increased numbers of CD137^+^ CD8^+^ T-cells, a marker for T-cell activation was found after the co-culture with day 20 SPZ (mean 12.3%; SD 6.9. Figure 7A–C) as compared to day 14 SPZ (mean 8.3%; SD 2.5). Similarly, an increased number of IFNγ^+^ CD8^+^ T-cells after co-culture with day 20 SPZ (mean 2.6%; SD 1.2. Figure 7A, D) compared to day 14 SPZ was found (mean 1.9%; SD 0.9). These data suggest that aging SPZ lose their ability to downregulate CD8^+^ T-cell activation.

To investigate whether the ability to downregulate CD8^+^ T-cells is unique to viable SPZ, the antigen presentation between MoMɸs, MoDCs and CD8^+^ T-cells after stimulation with dead SPZ, harvested and killed at the same ages was investigated. Dead SPZ overall showed less CD8^+^ T-cell activation as measured by CD137^+^ expression (day 14 2.4 fold decrease, day 20 2.9 fold decrease; Fig. 7A) and IFNγ^+^ production (day 14 1.6 fold decrease, day 20 2.8 fold decrease; Fig. 7B). Dead SPZ harvested at day 14 and day 20 showed comparable capacity to activate CD8^+^ T-cells as salivary gland extract (SGE) used as a negative control (CD137 day 14 p = 0.15, day 20 p = 1.0; IFNγ^+^ day 14 p = 0.78, day 20 p = 1.0) (Additional file 5A–C, Additional file 6). This makes us conclude that the differences of CD8^+^ T-cell downregulation are caused by viable SPZ.

In conclusion, the ratio of 14 days compared with 20 days post blood meal SPZ seems to be increased for uptake (1.85), expression and production of several anti- (PDL-1, 1.54; IL-10, 5.78) and pro-inflammatory (CD80, 1.33; IL-6, 3.82) markers and cytokines in MoMɸs, which was paralleled with an decrease of CD137^+^ (0.47) and IFNγ^+^ (0.73) CD8^+^ T-cells in co-culture experiments (Fig. 7C). These data suggest that aging SPZ lose the ability to downregulate CD8^+^ T-cell activation.

## Discussion

This study shows that old *P. falciparum* SPZ, harvested at 17–20 days post blood meal have decreased motility, with less motile SPZ and shorter tracks compared with day 14 *P*. *falciparum* SPZ. This is paralleled by a decrease of SPZ infectivity, SPZ uptake by macrophages and an decreased ability to downregulate CD8^+^ T-cells. These differences were not explained by a reduction in viability of SPZ of older ages, or a difference in ORC and ECAR metabolic states.

Co-culture of CD8^+^ T-cells with APCs stimulated with dead (heat killed) *P*. *falciparum* SPZ demonstrated that these age-related changes were not caused by dying SPZ. This resulted in the same level of CD8^+^ T-cell activation as for the negative SGE control and the opposite effect for day 20 SPZ. In addition, there were no changes in oxygen consumption rate between the younger day 14 and older day 20 *P*. *falciparum* SPZ. These findings indicate that day 20 SPZ are still alive and metabolically similarly active as their counterparts. It can thus be hypothesized that functional changes in the antigen expression and/or changes in motility ligands underlie the changing motility, infectivity and immunogenicity profile.

The remarkable alignment between the findings of SPZ motility and infectivity in vitro suggests that gliding motility is essential for SPZ to successfully infect. Whereas this is not very surprising, and has been reported before [35], the use of the in house custom made SMOOT software now allowed us to identify the subset of SPZ responsible for the subtle motility differences between SPZ at different ages. This led to the hypothesis that the SPZ subset with these specific characteristics (longer tracks, slower and more chaotic, e.g. cluster 1 motility SPZ, of which 49% day 14 SPZ) are the most infectious and therefore the decrease of their relative abundancy underlies decreasing infectivity at later ages.

The decrease in infectivity and regulatory potential of ageing *P*. *falciparum* SPZ may be particularly relevant to a better understanding of the infectious reservoir of naturally occurring Anopheline mosquitoes. Even though some *Anopheles* mosquitoes in nature can persist beyond 20 days, the data within this study suggest that these mosquitoes are likely much less infectious. In addition to the differences within mosquito salivary SPZ at different timepoints post blood meal, also *P. falciparum* oocyst SPZ were found to be less infectious and less motile as compared to *P. falciparum* salivary gland SPZ [10, 15, 36–38]. Genes essential to SPZ motility and infectivity, like CSP [39, 40] or TRAP [41], are specifically switched on just prior to SPZ release into the skin. Such switch in transcription patterns may be induced by changes in mosquito-derived proteins such as mosGILT, which is known to reduce SPZ infectivity [9]. Potentially such switches in gene expression may also be responsible for the age-related differences. Moreover, since *An. stephensi* is routinely used to generate whole SPZ vaccines, these findings are helpful for the further optimization of whole SPZ-based vaccination strategy.

The changing immunogenicity of SPZ at different ages may be particularly relevant for vaccine development. For example, the age of whole SPZ vaccines may affect their potency. Whole SPZ vaccines are one of the most successful vaccines so far even though their potency is somewhat lower in endemic sites [42–45]. Antigen presenting cells have been shown to play a critical role in the activation of CD8^+^ T-cells which are presumed to be one of the effector cells induced by whole SPZ vaccines [18–20]. This study demonstrated that APCs co-cultured with older SPZ are more likely to skew CD8^+^ T-cells towards a pro-inflammatory phenotype. The decreased regulatory potential of older SPZ begs the question of whether these aged SPZ may be more suited to induce effective malaria immunity. At the same time, whole SPZ vaccines critically rely on the infectivity of the vaccine SPZ, which may be much better at day 14. If the combination of changing infectivity and immunogenicity of aged SPZ in vitro will have similar impact on in vivo infectivity and immunogenicity is uncertain. Given the current limitations of animal models for assessing susceptibility to *P. falciparum* malaria and immunogenicity simultaneously, this question cannot be answered using pre-clinical models. Therefore, what the most optimal timepoint should be for whole SPZ vaccine manufacturing remains to be investigated. The in vitro data highlight the importance of age of SPZ used when addressing vaccine formulation and efficacy. Moreover, it also highlights the importance of standardization of mosquito age across research centres for pre-clinical and clinical whole-sporozoite malaria to allow for direct comparison of data outputs.

## Conclusion

This study aimed to get a better insight into functional changes in salivary gland SPZ and their effect on the human immune system. Day 17–20 SPZ were shown to have decreased motility, infectivity, SPZ uptake by macrophages and a decreased ability to downregulate CD8^+^ T-cells. Such insights are a first step towards a better understanding of salivary gland SPZ maturation, infectivity and regulatory potential.

### Supplementary Information

**Additional file 1.** SPZ number. A Number of SPZ at 14 days post blood meal (light blue), 17 days post blood meal (blue) and 20 days post blood meal (dark blue) from the same mosquito batch.

**Additional file 2.** Motility analysed by SMOOT. A Number frames per track per SPZ at day 14 in light blue, day 17 in blue and day 20 in dark blue. B Velocity per sporozoite track analysed by SMOOT. C Distance per sporozoite track in log scale analysed by SMOOT. D Tracks of day 14, day 17 and day 20 SPZ. E Angular dispersion of each track analysed by SMOOT. F Straightness index of each track analysed by SMOOT. Analysis using Mann Whitney U test. *: P=<0,05, **: P= <0,005, ***: P=<0,0005 and ****: P=<0,0001.

**Additional file 3. **Sporozoite metabolic activity. A OCR activity over time of SPZ of day 14 (light blue) or day 20 (dark blue). B ECAR activity over time of SPZ of day 14 (light blue) or day 20 (dark blue). C Oxygen consumption rate (OCR) and extracellular acidification rate (ECAR) shown in area under the curve (AUC). Day 14 SPZ (light blue) and day 20 SPZ (dark blue) D Basal energy metabolism calculated by dividing OCR by ECAR AUR values. Day 14 SPZ (light blue) and day 20 SPZ (dark blue). Analysis using Mann Whitney U test. *: P=<0.05, **: P= <0.005, ***: P=<0.0005 and ****: P=<0.0001.

**Additional file 4.** Immunogenicity. A Expression of CD163 after stimulation with lipopolysaccharide (LPS) as a positive control in black, salivary gland (SGE) negative control in grey, and SPZ(SPZ) used at day 14 light blue, day 17 blue and day 20 dark blue. Data shown as median fluorescence intensity (MFI) fold change relative to medium stimulated control. B Expression of CD209. **C** TNF cytokine production measured in the supernatant, 24h post stimulation.

**Additional file 5.** CD8^+^ T-cell responses. A Percentage of CD137^+^ CD8+ T-cells. CSP (black), day 14 and 20 SGE control (grey filled bar), day 14 SPZ (light blue), day 20 SPZ (dark blue), day 14 and 20 dead SPZ (grey striped bar). Data shown as fold change relative to medium stimulated control. **C** Percentage of IFNγ^+^ CD8+ T-cells . N=2, 6 donors. Analysis using Mann Whitney U test. *: P=<0,05, **: P= <0,005, ***: P=<0,0005 and ****: P=<0,0001.

**Additional file 6.** CD8^+^ T-cells co-culture. A Gating strategy of CD137^+^ and IFNγ^+^ CD8^+^ T-cells after stimulation with medium, CSP, SPZ day 14, SPZ day 20.

## Data Availability

The datasets supporting the conclusion of this article are included within the article.
