# Supplementary figures and images for: Ageing of Plasmodium falciparum malaria sporozoites alters their motility, infectivity and reduces immune activation in vitro
Source: Malar J. 2024 Apr 19;23:111. doi: 10.1186/s12936-024-04946-7 (PMC11027264; doi:10.1186/s12936-024-04946-7)

## Slide 1
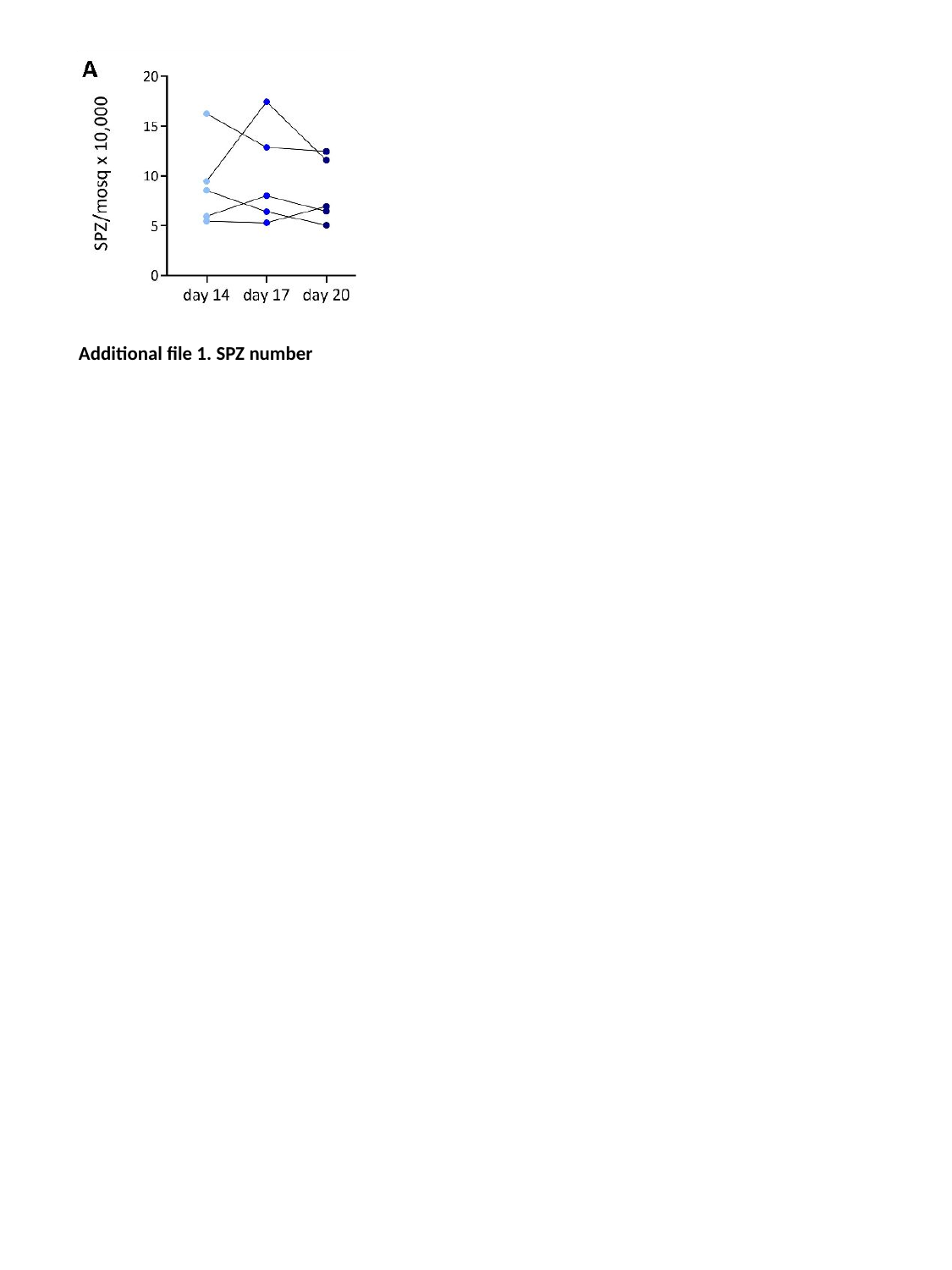

Additional file 1. SPZ number

Supplement: Supplementary file 1 — Additional file 1. SPZ number. A Number of SPZ at 14 days post blood meal (light blue), 17 days post blood meal (blue) and 20 days post blood meal (dark blue) from the same mosquito batch. [file 12936_2024_4946_MOESM1_ESM.pptx]

## Slide 1
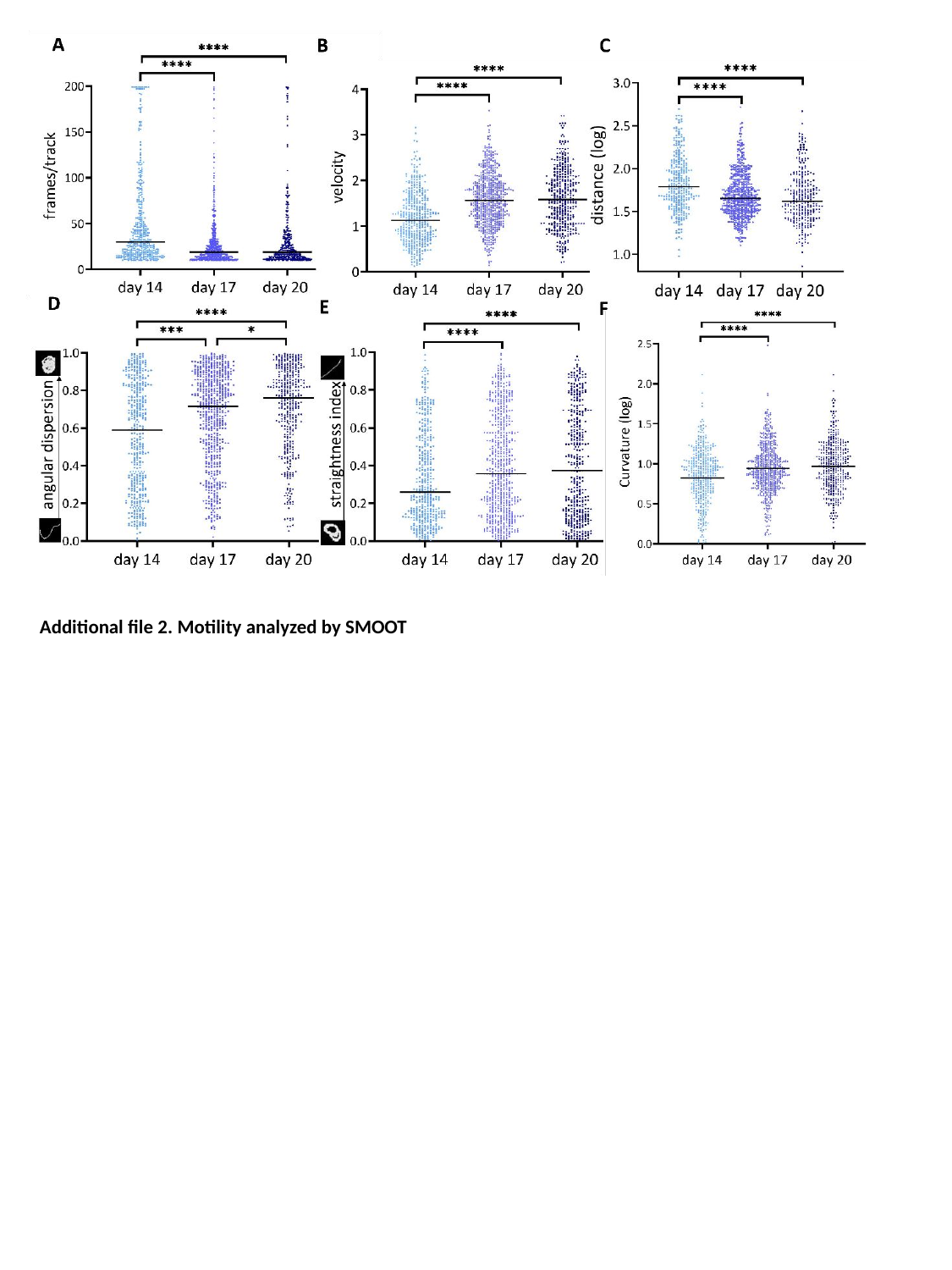

Additional file 2. Motility analyzed by SMOOT

Supplement: Supplementary file 2 — Additional file 2. Motility analysed by SMOOT. A Number frames per track per SPZ at day 14 in light blue, day 17 in blue and day 20 in dark blue. B Velocity per sporozoite track analysed by SMOOT. C Distance per sporozoite track in log scale analysed by SMOOT. D Tracks of day 14, day 17 and day 20 SPZ. E Angular dispersion of each track analysed by SMOOT. F Straightness index of each track analysed by SMOOT. Analysis using Mann Whitney U test. *: P=<0,05, **: P= <0,005, ***: P=<0,0005 and ****: P=<0,0001. [file 12936_2024_4946_MOESM2_ESM.pptx]

## Slide 1
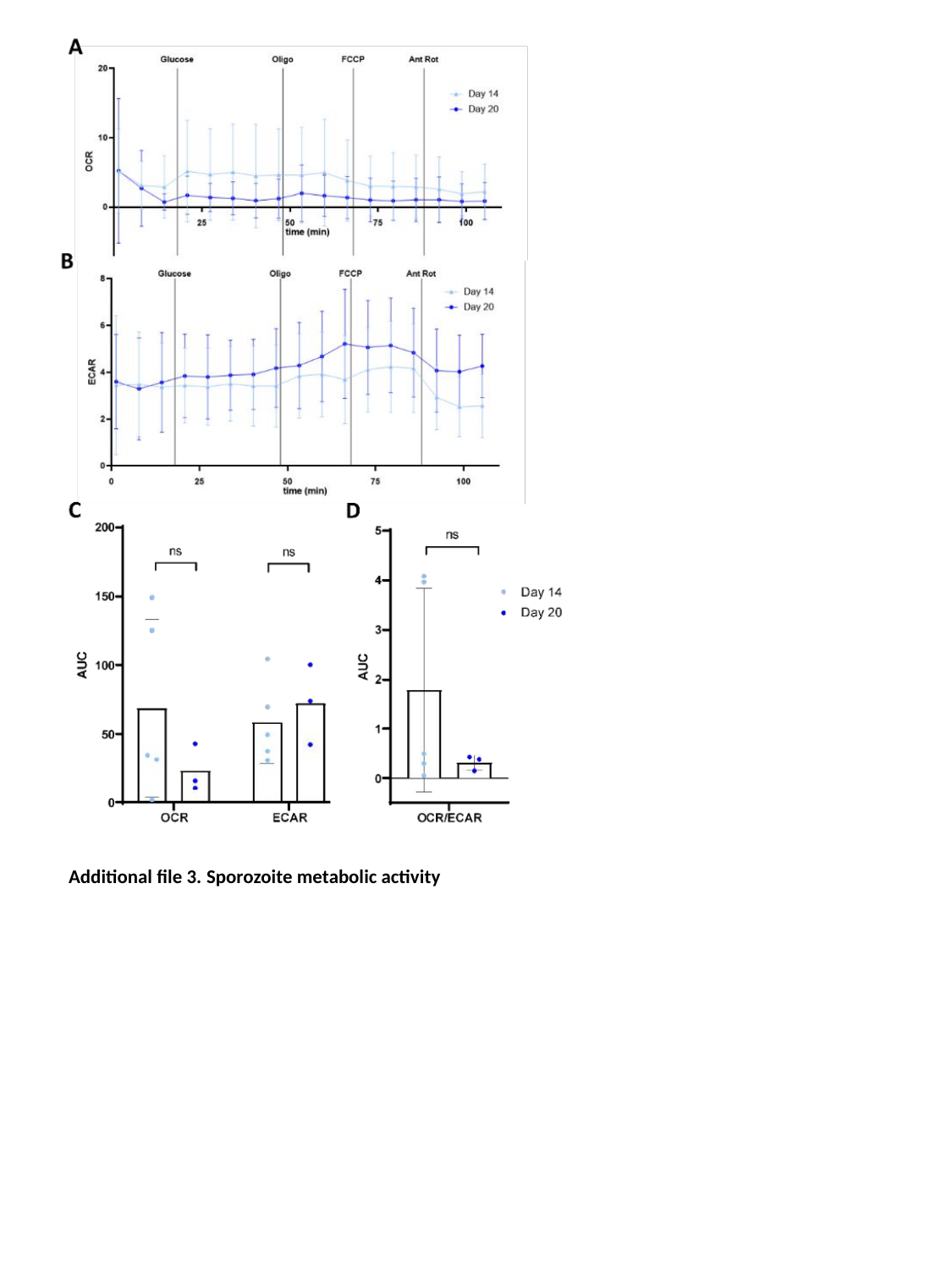

Additional file 3. Sporozoite metabolic activity

Supplement: Supplementary file 3 — Additional file 3. Sporozoite metabolic activity. A OCR activity over time of SPZ of day 14 (light blue) or day 20 (dark blue). B ECAR activity over time of SPZ of day 14 (light blue) or day 20 (dark blue). C Oxygen consumption rate (OCR) and extracellular acidification rate (ECAR) shown in area under the curve (AUC). Day 14 SPZ (light blue) and day 20 SPZ (dark blue) D Basal energy metabolism calculated by dividing OCR by ECAR AUR values. Day 14 SPZ (light blue) and day 20 SPZ (dark blue). Analysis using Mann Whitney U test. *: P=<0.05, **: P= <0.005, ***: P=<0.0005 and ****: P=<0.0001. [file 12936_2024_4946_MOESM3_ESM.pptx]

## Slide 1
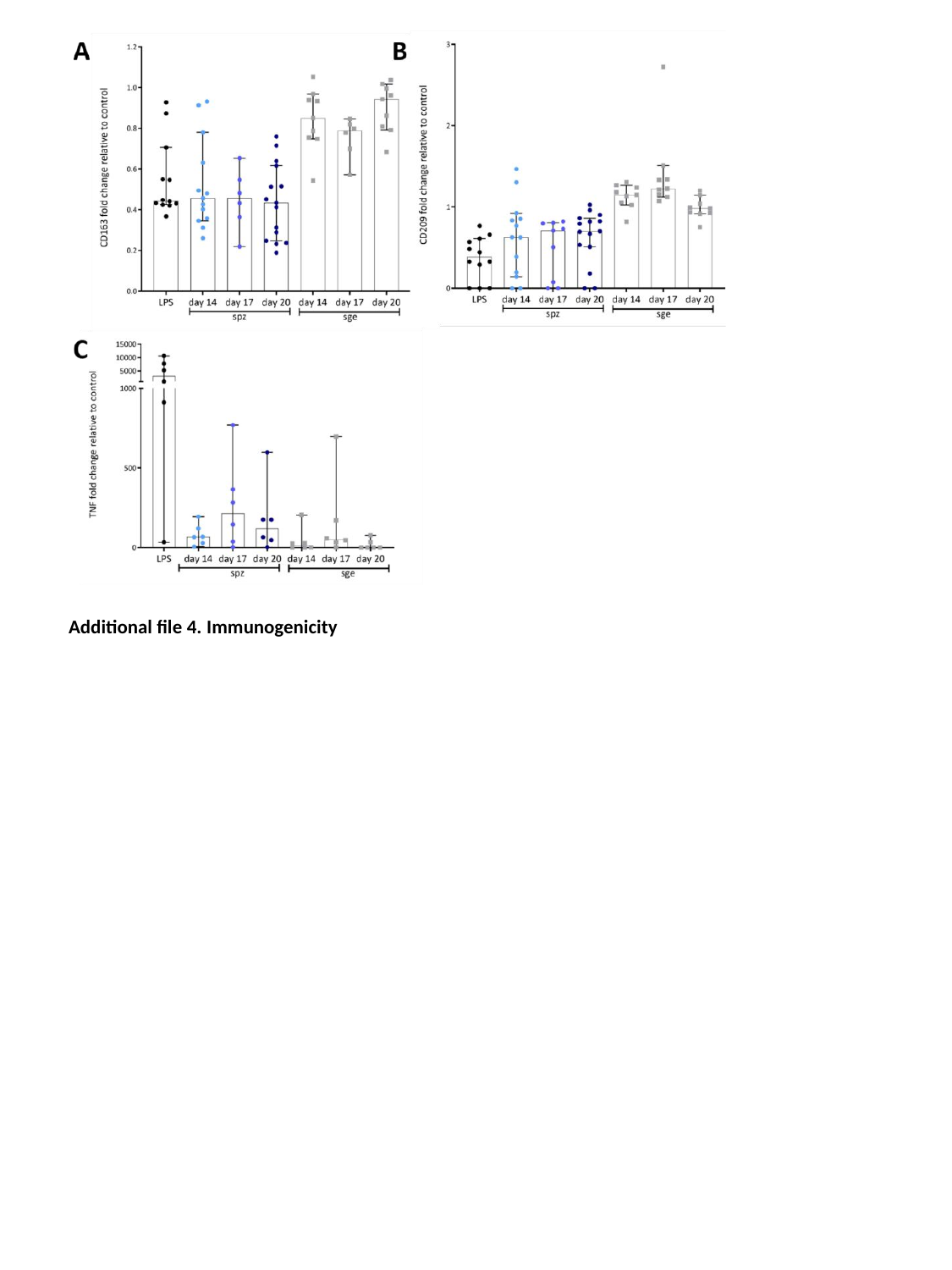

Additional file 4. Immunogenicity

Supplement: Supplementary file 4 — Additional file 4. Immunogenicity. A Expression of CD163 after stimulation with lipopolysaccharide (LPS) as a positive control in black, salivary gland (SGE) negative control in grey, and SPZ(SPZ) used at day 14 light blue, day 17 blue and day 20 dark blue. Data shown as median fluorescence intensity (MFI) fold change relative to medium stimulated control. B Expression of CD209. C TNF cytokine production measured in the supernatant, 24h post stimulation. [file 12936_2024_4946_MOESM4_ESM.pptx]

## Slide 1
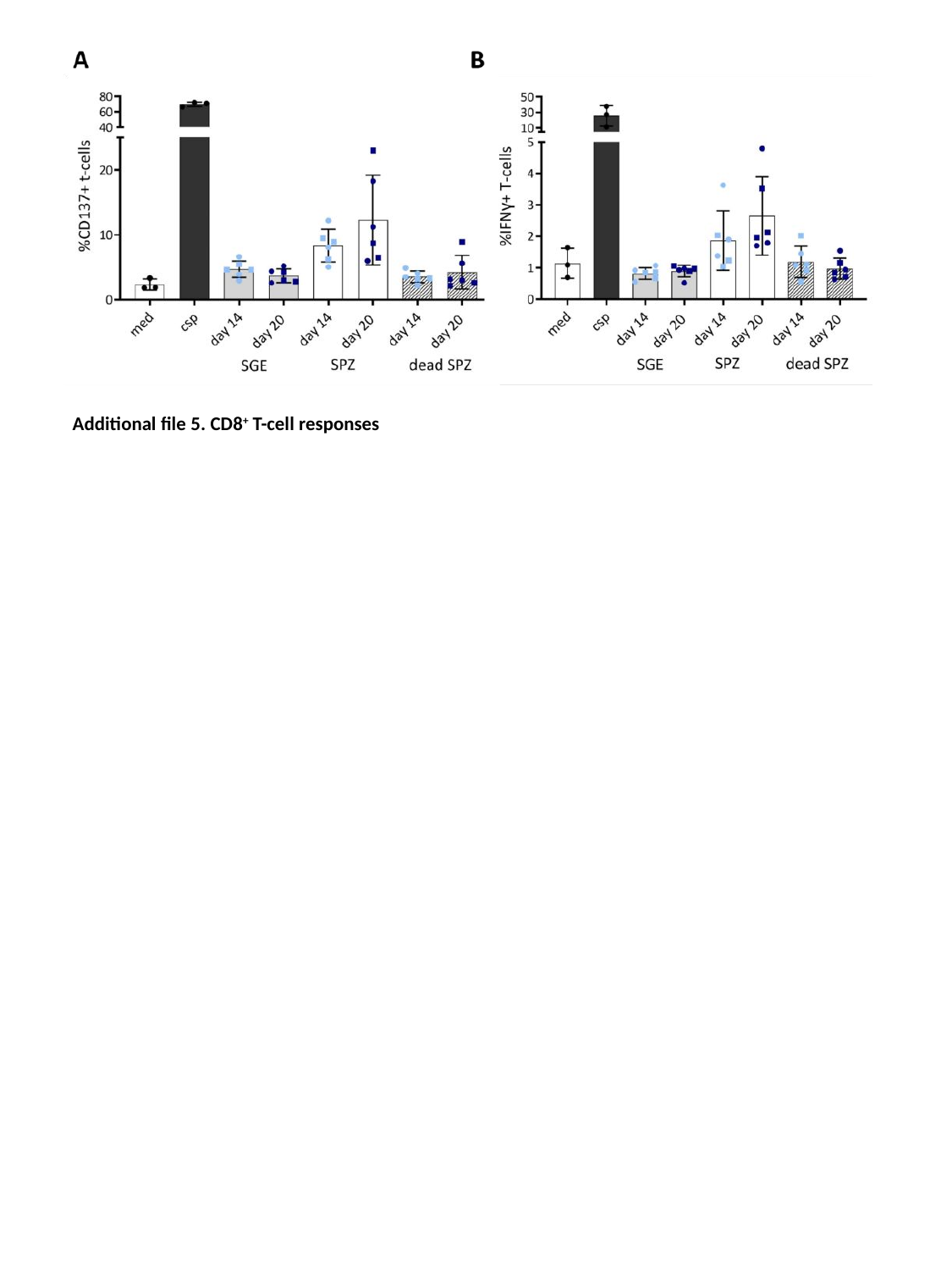

Additional file 5. CD8+ T-cell responses

Supplement: Supplementary file 5 — Additional file 5. CD8+ T-cell responses. A Percentage of CD137+ CD8+ T-cells. CSP (black), day 14 and 20 SGE control (grey filled bar), day 14 SPZ (light blue), day 20 SPZ (dark blue), day 14 and 20 dead SPZ (grey striped bar). Data shown as fold change relative to medium stimulated control. C Percentage of IFNγ+ CD8+ T-cells . N=2, 6 donors. Analysis using Mann Whitney U test. *: P=<0,05, **: P= <0,005, ***: P=<0,0005 and ****: P=<0,0001. [file 12936_2024_4946_MOESM5_ESM.pptx]

## Slide 1
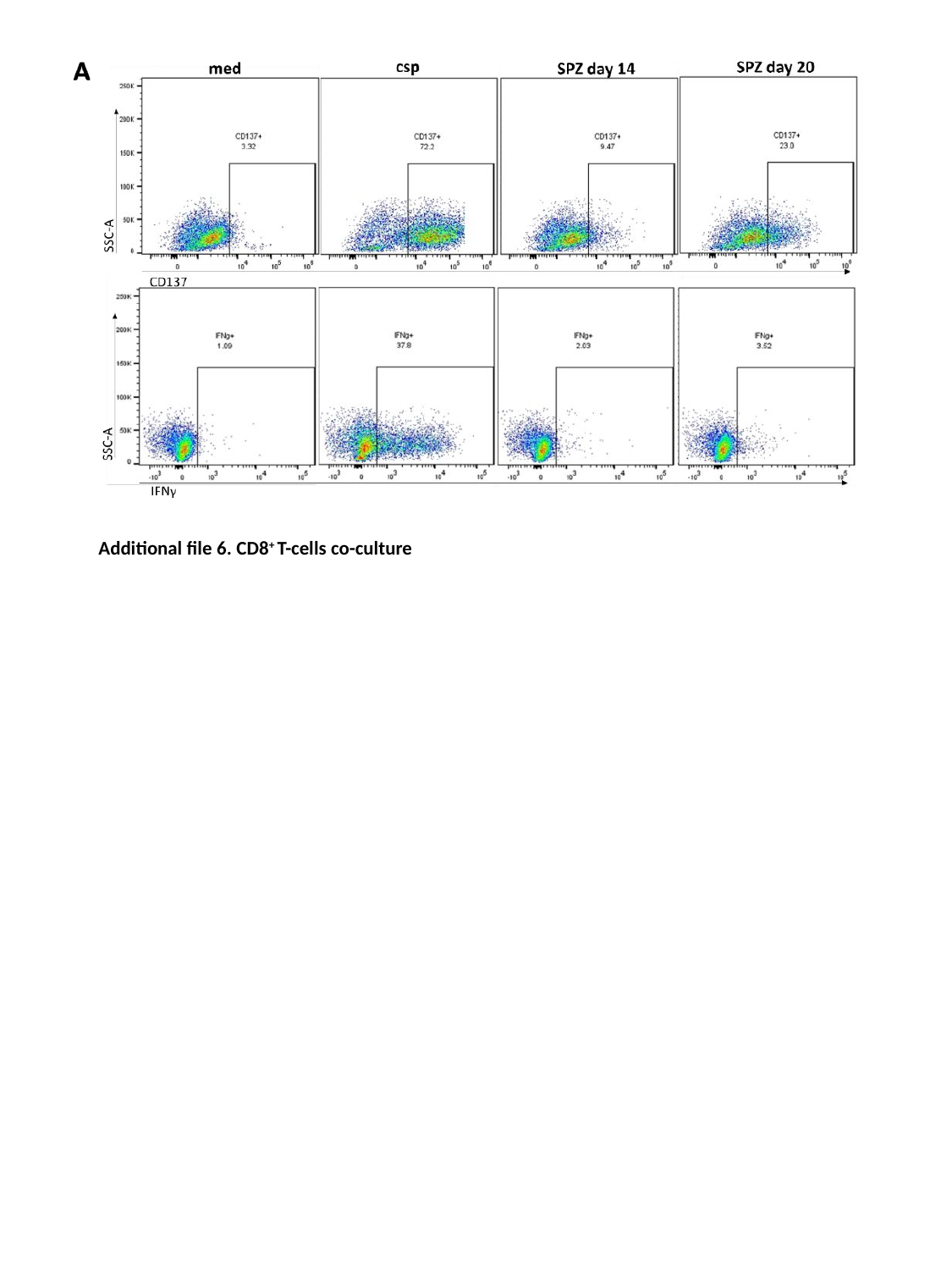

Additional file 6. CD8+ T-cells co-culture

Supplement: Supplementary file 6 — Additional file 6. CD8+ T-cells co-culture. A Gating strategy of CD137+ and IFNγ+ CD8+ T-cells after stimulation with medium, CSP, SPZ day 14, SPZ day 20. [file 12936_2024_4946_MOESM6_ESM.pptx]
